# A simplified combined index of structure and function for detecting and staging glaucomatous damage

**DOI:** 10.1038/s41598-021-82756-6

**Published:** 2021-02-04

**Authors:** Zhichao Wu, Felipe A. Medeiros

**Affiliations:** 1 Duke Eye Center, Department of Ophthalmology, Duke University School of Medicine, 2351 Erwin Rd, Durham, NC 27705 USA; 2 Department of Ophthalmology, University of California, San Diego, La Jolla, CA USA; 3 Centre for Eye Research Australia, Royal Victorian Eye and Ear Hospital, East Melbourne, Australia; 4 Ophthalmology, Department of Surgery, The University of Melbourne, Melbourne, Australia

**Keywords:** Eye diseases, Optic nerve diseases

## Abstract

Glaucomatous damage results in characteristics structural and functional changes on optical coherence tomography (OCT) imaging and standard automated perimetry (SAP) testing. The clinical utility of these measures differs based on disease severity, as they are evaluated along different measurement scales. This study therefore sought to examine if a simplified combined structure–function index (sCSFI) could improve the detection and staging of glaucomatous damage, compared to the use of average retinal nerve fiber layer thickness (RNFL) measurements from OCT and mean deviation (MD) values from SAP alone, and also an estimated retinal ganglion cell counts (eRGC) measure derived using empirical formulas described previously. Examining 577 eyes from 354 participants with perimetric glaucoma and 241 normal eyes from 138 healthy participants, we found that the sCSFI performed significantly better than average RNFL, MD and eRGC count for discriminating between glaucoma and healthy eyes (*P* ≤ 0.008 for all). The sCSFI also performed significantly better than RNFL and eRGC count at discriminating between different levels of visual field damage in glaucoma eyes (*P* < 0.001 for both). These findings highlight the clinical utility of combining structural and functional information for detecting and staging glaucomatous damage using the simplified index developed in this study.

## Introduction

Glaucoma is a condition characterized by the progressive degeneration of retinal ganglion cells (RGCs) and their axons, resulting in a thinning of the neuroretinal rim and retinal nerve fiber layer (RNFL). Such structural changes are often accompanied by visual function losses that may lead to disability^1^. Clinical measures of structural and functional damage in glaucoma are typically evaluated on different measurement scales and their accuracy and utility has been shown to depend on the stage of the disease^2–5^. For instance, RNFL loss as measured by optical coherence tomography (OCT) is often detected before changes in visual sensitivity on standard automated perimetry (SAP) are apparent^6–14^. On the other hand, in more severe disease, RNFL parameters have diminished value for staging or detecting disease progression due to measurement floors^15,16^.

Due to their different characteristics, frequent disagreements are often seen when structural and functional tests are used to detect glaucoma or monitor its progression, and that can be confusing to practitioners. However, the differences in the clinical accuracy and utility of structural and functional measures can be exploited to improve detection and staging of glaucomatous damage. We previously proposed a new parameter that sought to do this through combining RNFL thickness measurements from OCT imaging with visual sensitivity from SAP using a novel weighted averaging scheme^2^, after transforming both parameters into estimates of RGCs using empirically derived formulas^4^. Previous studies have demonstrated the clinical advantages of such a parameter when compared to isolated structural and visual function measures, including an improved detection of the disease and its progression^2,17–24^.

However, two recent studies have cautioned against some assumptions of the empirical formulas used for estimating RGC counts employed in the combined index, highlighting the disagreement between estimates of RGCs with SAP and OCT and the discrepancy with histological data^25,26^. Nevertheless, both studies recognized that the issue of estimating RGCs differed from the clinical value of combining structural and visual function measures^25,26^, and suggested evaluating the performance of a combined metric without estimating RGCs^25^. We thus propose in this study a simplified index for combining structural and functional measurements that does not rely on empirical formulas for estimating RGC counts. We investigated its ability to discriminate glaucomatous from healthy eyes as well its performance in staging the disease.

## Results

### Participant characteristics

A total of 2,217 tests from 577 eyes of 354 participants with glaucoma were included, and these participants were on average 68 ± 11 years old (range 31–96 years old). A total of 582 tests from 241 normal eyes of 138 participants were also included in this study, and these participants were on average 53 ± 16 years old (range 21–92 years old). The characteristics of these participants at their first visit are summarized in Table 1.

**Table 1:** Characteristics of the eyes included in this study at the first visit.

Parameter	Glaucoma (*n* = 577 eyes)	Healthy (*n* = 241 eyes)
MD (dB)	− 2.28 (− 6.09 to − 0.48)	− 0.05 (− 0.83 to 0.82)
PSD (dB)	2.42 (1.70 to 7.74)	1.51 (1.32 to 1.75)
RNFL thickness (μm)	74 ± 13	93 ± 10
eRGC count (× 1000 cells)	624 ± 222	1020 ± 181
sCSFI (%)	65 ± 29	98 ± 11

Data are presented either as median (interquartile range) or the mean ± standard deviation; MD, mean deviation; PSD, pattern standard deviation; RNFL, retinal nerve fiber layer; eRGC Count, estimated retinal ganglion cell count; sCSFI, simplified combined structure–function index.

### Performance for detecting glaucoma

The performance of each parameter for discriminating between the perimetric glaucoma, all glaucoma eyes and glaucoma eyes with MD ≥ − 6 dB from normal eyes was evaluated, and the results are shown in Table 2. For perimetric glaucoma, the performance of an estimated retinal ganglion cell (eRGC) count and a simplified combined structure–function index (sCSFI; area under the receiver operating characteristic curve [AUC] = 0.94 and 0.95 respectively) measure (with the methods for their calculations described in the Methods section) were both superior compared to average RNFL thickness (AUC = 0.90; *P* < 0.001) from OCT imaging and mean deviation (MD) from SAP testing (AUC = 0.90; *P* ≤ 0.011). When compared with each other, the performance of the sCSFI for discriminating between healthy and glaucoma eyes was superior compared to the eRGC parameter (*P* = 0.001). Similar findings of a better performance of the eRGC count and sCSFI parameters for discriminating all glaucoma eyes and early glaucoma eyes (MD ≥ − 6 dB) from healthy eyes compared to average RNFL thickness and MD were observed (*P* ≤ 0.007). The sCSFI also similarly performed better than the eRGC parameter for discriminating all glaucoma eyes and early glaucoma eyes (*P* ≤ 0.009).

**Table 2:** Areas under the receiver operating characteristic curves (AUC) and sensitivity at 95% and 80% specificity for the parameters evaluated in this study.

Parameter	*P* value against			Sensitivity (%) at	
	AUC (SE)	eRGC Count	sCSFI	95% Specificity	80% Specificity
**Perimetric glaucoma** (n = 392 eyes)					
MD	0.91 (0.01)	0.011*	< 0.001*	73	84
RNFL thickness	0.90 (0.02)	< 0.001*	< 0.001*	70	83
eRGC count	0.94 (0.01)	–	0.001*	79	90
sCSFI	0.95 (0.01)	0.001*	–	83	92
**All glaucoma** (n = 577 eyes)					
MD	0.79 (0.02)	< 0.001*	< 0.001*	51	65
RNFL thickness	0.83 (0.02)	0.002*	< 0.001*	56	72
eRGC count	0.86 (0.02)	–	0.008*	60	76
sCSFI	0.87 (0.02)	0.008*	–	64	79
**Glaucoma MD ≥ **− **6 dB** (n = 442 eyes)					
MD	0.71 (0.02)	< 0.001*	< 0.001*	33	52
RNFL thickness	0.78 (0.02)	0.007*	< 0.001*	43	63
eRGC count	0.81 (0.02)	–	0.009*	46	68
sCSFI	0.83 (0.02)	0.009*	–	52	72

Glaucoma was defined on the basis of the optic nerve appearance on masked grading, and perimetric glaucoma was further considered to present based on a history of ≥ 3 consecutive abnormal visual field test results (see “Methods” section); SE, standard error; MD, mean deviation, RNFL, retinal nerve fiber layer, eRGC Count, estimated retinal ganglion cell count, sCSFI, simplified combined structure–function index; * = statistically significant at *P* < 0.05.

### Magnitude of measured abnormality (deviation from normal)

**Figure 1:**
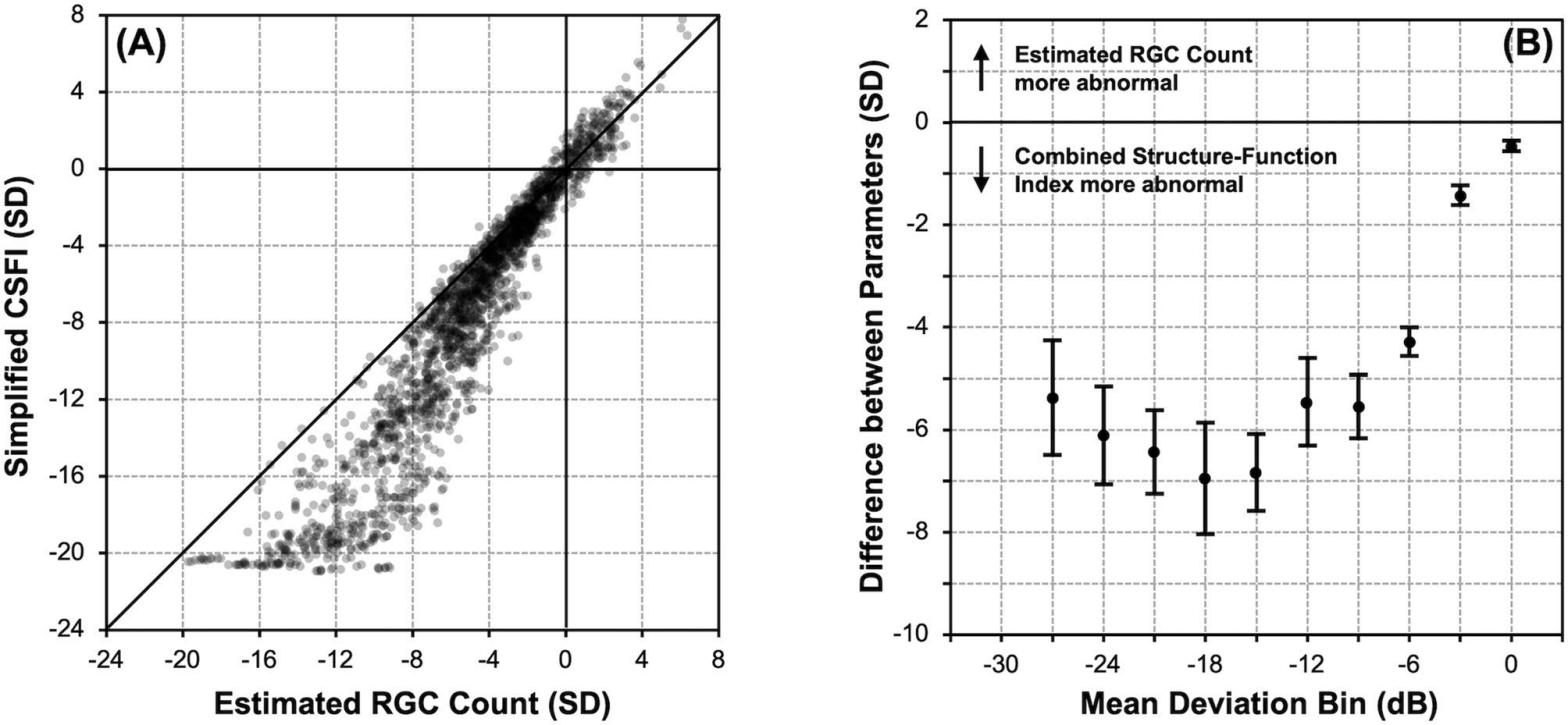
(**A**) Scatterplot of the Z-scores (standard deviations [SD] away from the normal cohort) of the simplified combined structure–function index (sCSFI) against the estimated retinal ganglion cell (RGC) count for the eyes with glaucoma. (**B**) The difference in Z-scores between the sCSFI and estimated RGC count across visual field mean deviation bins (including at least 100 samples), illustrating how the sCSFI captures a significantly greater magnitude of abnormality (or deviation from normal) than the estimated RGC for all bins (P < 0.001).

The Z-scores (number of standard deviations away from normal) of the sCSFI and eRGC count of all the glaucoma eyes were determined using age-adjusted values of the normal eyes, and these are plotted in Fig. 1A. The difference between the Z-scores between the sCSFI and eRGC were then compared across visual field MD bins as shown in Fig. 1B, demonstrating that the sCSFI detected a significantly greater extent of abnormality (or deviation from normal) for all bins (*P* < 0.001).

### Discriminatory ability between different levels of visual field loss

The eRGC count and sCSFI parameter both performed better at discriminating between glaucoma eyes with early and moderate visual field loss (AUC = 0.95 and 0.99 respectively) compared to the average RNFL thickness (AUC = 0.77; *P* < 0.001 for both), but the sCSFI also performed better than the eRGC for this task (*P* < 0.001). The eRGC count and sCSFI performed similarly at discriminating between glaucoma eyes with moderate and advanced visual field loss (AUC = 0.89 and 0.92 respectively; *P* = 0.240), and also better than average RNFL thickness (AUC = 0.70; *P* < 0.001 for both). The ability to discriminate between glaucoma eyes across different levels of glaucomatous visual field loss at smaller intervals (MD in 3-dB bins) was evaluated by estimating the proportion of variance explained by each parameter during ordinal logistic regression analyses (i.e. ability to correctly predict the category, summarized using the pseudo-*R*^2^ measure of the proportion of variance explained). The *R*^2^ for the sCSFI, eRGC and average RNFL thickness were 0.82, 0.74 and 0.29 respectively, and were all significantly different from each other (*P* < 0.001 for all comparisons), which highlights the improved ability of the sCSFI to discriminate between different levels of visual field damage; these findings are summarized in Table 3.

**Table 3:** Discriminatory ability of each parameter for different levels of visual field mean deviation (MD) loss for the glaucoma eyes.

Parameter	AUC, comparisons between		Pseudo-*R*^2^
	Early versus moderate	Moderate versus advanced	
RNFL thickness	0.77 (0.04)	0.70 (0.07)	0.29 (0.01)
eRGC count	0.95 (0.01)*	0.89 (0.02)*	0.74 (0.01)*
sCSFI	0.99 (0.00)^#^	0.92 (0.02)*	0.82 (0.01)^#^

Comparisons were performed between glaucoma eyes that had early (MD > − 6 dB), moderate (MD − 6 to − 12 dB) and advanced (MD < − 12 dB) visual field loss. AUC, area under the receiver operating characteristic curve; RNFL, retinal nerve fiber layer; eRGC Count, estimated retinal ganglion cell count; sCSFI, simplified combined structure–function index; * = better compared to RNFL thickness at *P* < 0.001; ^**#**^ = better compared to RNFL thickness and eRGC count at *P* < 0.001.

### Clinical relevance and examples

**Figure 2:**
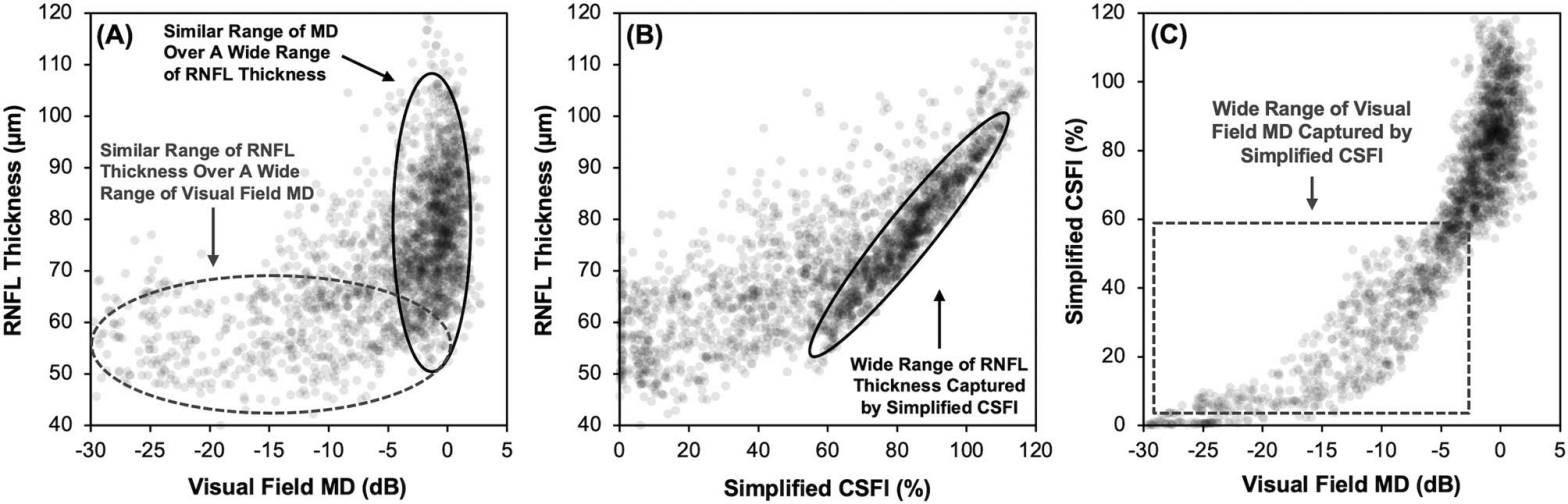
Scatterplot of the average retinal nerve fiber layer (RNFL) thickness plotted against the visual field mean deviation (MD; **A**) and the simplified combined structure–function index (sCSFI; **B**), and the sCSFI plotted against the visual field MD (**C**).

To illustrate the clinical relevance of the sCSFI, the average RNFL thickness of all eyes within the validation cohort were plotted against visual field MD, which demonstrated a non-linear relationship between these two parameters. This means that glaucoma eyes can be characterized by having a wide range of RNFL thickness when the MD is relatively normal, but that the RNFL thickness remains within a small range over a wide range of visual field MD (Fig. 2A). However, the wide range of RNFL thicknesses for the glaucomatous eyes with relatively normal visual fields is captured by the sCSFI (Fig. 2B), and the wide range of abnormal visual field MD is also captured by the very same index (Fig. 2C). This highlights the ability of the sCSFI to characterize the disease over its entire spectrum.

**Figure 3:**
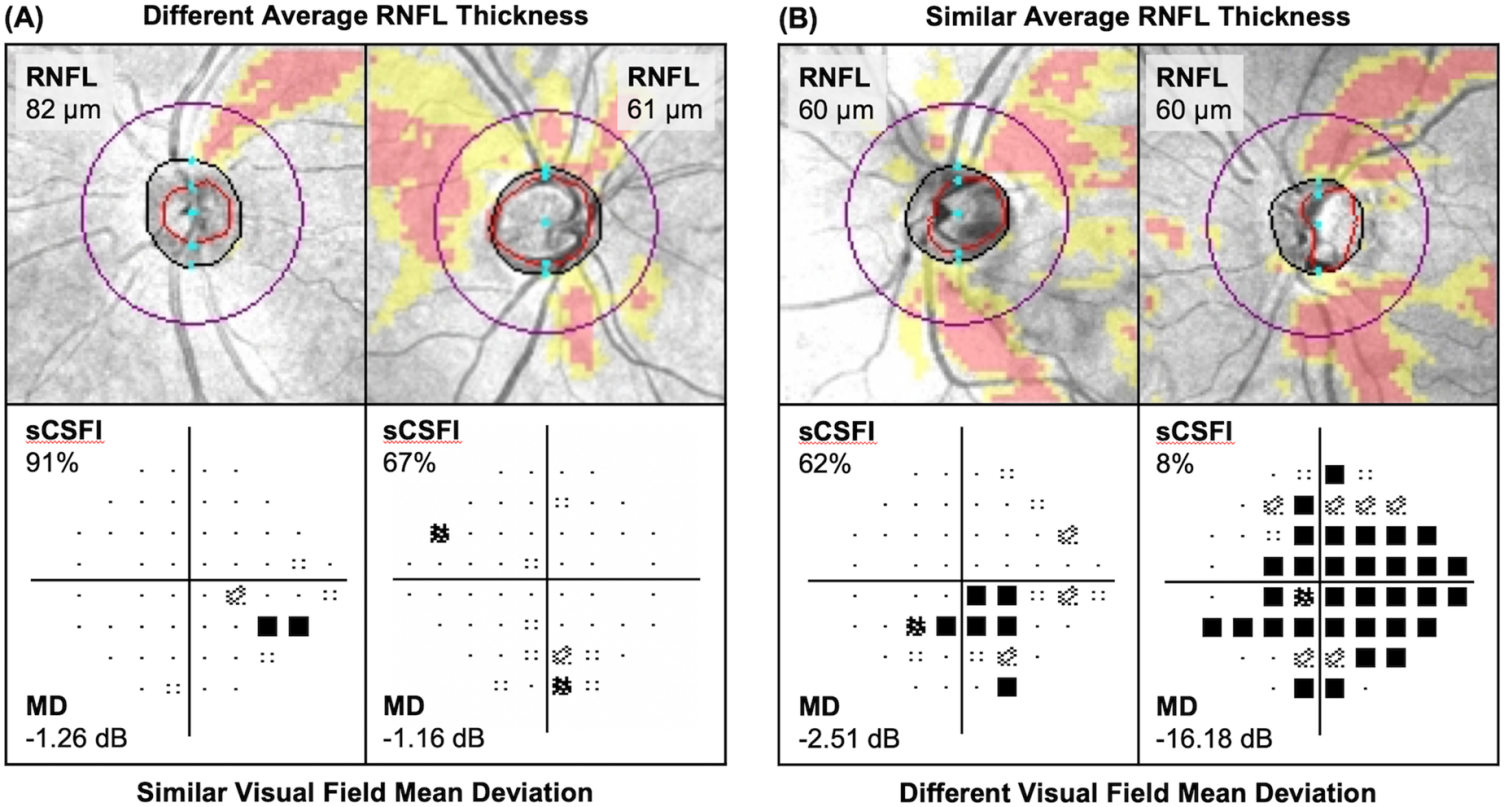
Examples of how simplified combined structure function index (sCSFI) captures the extent of glaucomatous damage across the spectrum of the disease. (**A**) In the first example, two eyes with similar visual field mean deviation (MD) show markedly different average retinal nerve fiber layer (RNFL) thicknesses, but this difference was apparent on the sCSFI. (**B**) In the second example, two eyes with similar average RNFL thicknesses show markedly different visual field MD, which was also different on the sCSFI.

Examples of the ability for the sCSFI to capture glaucomatous damage across the entire disease spectrum are shown in Fig. 3. In the first example (Fig. 3A), two glaucoma eyes from a 77- and 60-year-old participants with similar visual field MD (− 1.26 and − 1.16 dB respectively) can have markedly different RNFL thicknesses (82 and 61 μm respectively), although this difference was apparent on the sCSFI (91 and 67% respectively). In the second example (Fig. 3B), two glaucoma eyes from a 73- and 83-year-old participants with similar average RNFL thicknesses (60 μm for both respectively) can have markedly different visual field MD (− 2.51 and − 16.18 dB), which was also apparent on the sCSFI (62 and 8% respectively).

## Discussion

This study demonstrated that a simplified index that combines RNFL thickness measurements on OCT imaging and visual sensitivity measurements on SAP captured the utility of each measure for the detection and staging the magnitude of glaucomatous damage across the entire spectrum of the disease. This was a similar case for the eRGC count parameter, although the sCSFI had an improved performance at detecting and staging the disease. In addition, the sCSFI does not rely on assumptions needed to obtain estimate of RGC counts. These findings highlight the utility of combining structural and visual function parameters for the clinical management of glaucoma.

In addition to the ability to detect a diseased state, the ability for a parameter to stage the severity of glaucomatous damage is also crucial. Most glaucoma staging systems are based on the extent of visual field abnormality^27^, although some are based on the extent of structural changes (such as optic disc damage^28^). Although the ability to stage disease severity can be evaluated by examining the AUC between pairs of disease severities, it can also be summarized by examining the ability of a parameter to correctly predict the degree of glaucomatous visual field damage at small intervals (such as visual field MD in 3-dB bins) and expressed using the pseudo *R*^2^ statistic^29^. We found that the average RNFL thickness was a poor predictor of visual field MD between eyes (pseudo *R*^2^ = 0.25), but that the sCSFI and eRGC count both performed significantly better (pseudo *R*^2^ = 0.85 and 0.74 respectively) as a result of incorporating visual field information.

These findings of the improved ability to detect and stage glaucomatous damage with the sCSFI compared to either structural or functional measures in isolation are apparent when examining the relationship between the results of the two clinical tests. For instance, the RNFL thickness can vary widely when visual field MD is relatively normal, but the RNFL thickness values reach a measurement floor^5,15,16^ while visual field sensitivities can still span a wide range of values. Instead, the sCSFI captures these differences across the entire spectrum of the disease, in a similar manner to the eRGC count parameter as we have demonstrated in our previous studies^2,3^. As such, the sCSFI provides an improved means to detect and stage glaucomatous damage across the entire disease spectrum, making use of the structural and functional measures at their optimal clinical accuracies to provide a single and intuitive estimate of disease severity.

The improved ability to detect and stage the glaucomatous severity with the sCSFI compared to the eRGC count parameter may be attributed to several factors. Although a similar weighted averaging system based on the level visual field damage was used for both parameters^2^, the sCSFI performs this averaging for mean TD values between 0 and − 10 dB for each quadrant, as opposed to averaging between MD values of 0 and − 30 dB for the eRGC count. This was performed since the RNFL thickness often becomes asymptotic when the mean TD is below − 10 dB^5^. In fact, the sCSFI performed similarly to the eRGC count parameter when the averaging was performed for mean TD values between 0 and − 30 dB (data not shown). In addition, the OCT component of the sCSFI was not transformed based on any assumptions about its relationship with visual sensitivity, such as seeking to account for age-related remodeling of the RNFL axonal density used for the eRGC count^4^, which may have introduced additional variability. Nevertheless, the utility of both combined indices support previous reports on the clinical value of the eRGC count parameter^2,17–22^, and suggests that these benefits are likely to be observed for the sCSFI as well; future studies are required to confirm this.

The sCSFI provides useful information for the clinical management of glaucoma, by providing a single intuitive-to-interpret, age-adjusted approximation of the percentage of glaucomatous damage across its entire dynamic range. However, a limitation of this study was the inability to assess its performance at staging disease severity, since there are currently no readily accepted and robust measures of this across the entire disease spectrum apart from using visual field measures, which are used as part of calculating the sCSFI. Therefore, future studies are now needed to examine the relationship between the sCSFI and an independent measure of functional disability, so that the interpretation of this measure in relation to its impact on outcomes relevant to a patient can be established^1^. To further establish the clinical utility of the sCSFI, future studies are now also required to examine its performance for capturing disease progression in glaucoma. The methods developed in this study to combine the OCT and visual field measures also provide a useful framework for future studies to explore whether other clinical parameters or measures that could be combined to further improve their use for detecting and staging glaucomatous damage. For example, neuroretinal thickness measurements from the macular region could be combined with central visual field sensitivity measurements to further improve the characterization of early glaucomatous macular damage. Functional imaging techniques (e.g. OCT angiography) and novel visual function and electrophysiological tests could also be combined with current clinical tests to further improve the detection and characterization of glaucomatous damage.

In conclusion, both the sCSFI performed better than each of the structural and visual function parameters used in isolation at detecting and staging the extent of glaucomatous damage, underscoring the clinical utility of combining these parameters for the clinical management of glaucoma. The methods developed in this study also provides a framework that can be used in future studies to explore whether other clinical measures could be combined to further improve the detection and characterization of glaucomatous damage.

## Methods

### Participants

Participants included in this study were involved in a prospective longitudinal study that evaluated structural and visual function changes in glaucoma. Institutional review board approval by the University of California, San Diego was provided for this study, and it was conducted in adherence with the Declaration of Helsinki and the Health Insurance Portability and Accountability Act. Written informed consent was obtained from all participants after the test procedures were explained.

In this study, a comprehensive ophthalmologic examination was performed for all participants at baseline. This included a medical history review, visual acuity and visual field testing, slit-lamp biomicroscopy and fundus examination, stereoscopic optic disc photography, measurements of intraocular pressure and gonioscopy. At baseline and each follow-up visit, visual field testing and OCT imaging was also performed.

All participants were required to be 18 years or older in this study and were excluded if they presented any other ocular or systemic disease that could affect the optic nerve or the visual field. Eyes of eligible participants were included if they had a best-corrected visual acuity of 20/40 or better and open angles on gonioscopy.

In this study, eyes were considered to have glaucoma on the basis of masked grading of the optic nerve appearance on stereophotographs, using methods described previously^30,31^. Eyes were then further considered to have perimetric glaucoma based on having a history of ≥ 3 consecutive abnormal visual field test results (defined as having a pattern standard deviation value at *P* < 0.05, or a glaucoma hemifield test outside normal limits)^13^. A control group was included in this study and consisted of subjects who were recruited from the general population and who had normal ophthalmological examination and intraocular pressures below 22 mm Hg.

### Visual field testing

In this study, visual field testing was performed using the Swedish Interactive Thresholding Algorithm Standard 24–2 strategy on the Humphrey Field Analyzer II-i (Carl Zeiss Meditec, Inc.; Dublin, CA, USA). Tests were considered unreliable if there was > 33% fixation losses or false negative errors (except false negative errors when the visual field MD was less than − 12 dB), or > 15% of false positive errors^32^. Visual field tests were reviewed for the artifacts such as fatigue or learning effect, inattention, inappropriate fixation, rim or eyelid artifacts, or any evidence that the visual field results were caused by another disease apart from glaucoma (e.g. homonymous hemianopia)^33^. Unreliable tests and those with artifacts were excluded from this study.

### Optical coherence tomography imaging

OCT imaging in this study was performed using the Cirrus HD-OCT (Carl Zeiss Meditec, Inc.; Dublin, CA, USA). This device uses a superluminescent diode with a central wavelength of 840 nm, and it obtains 27,000 A-scans per second and scans have an axial resolution of 5-μm. An optic disc volume scan was performed, and it consisted of 200 × 200 A-scans that covered a 6 × 6 mm centered on the optic disc^34^. From this scan, a 3.46 mm diameter circle consisting of 256 A-scans automatically centered on the optic discs was derived, and this scan was used to calculate the peripapillary RNFL thickness. All OCT volume scans were reviewed for the presence of improper centering of the derived circle scan (and it was manually adjusted if it was not properly centered) and presence of movement artifacts. Scans were excluded if movement artifacts were present, and also if the signal strength score was not ≥ 7^34^.

### Simplified combined-structure function index

**Figure 4:**
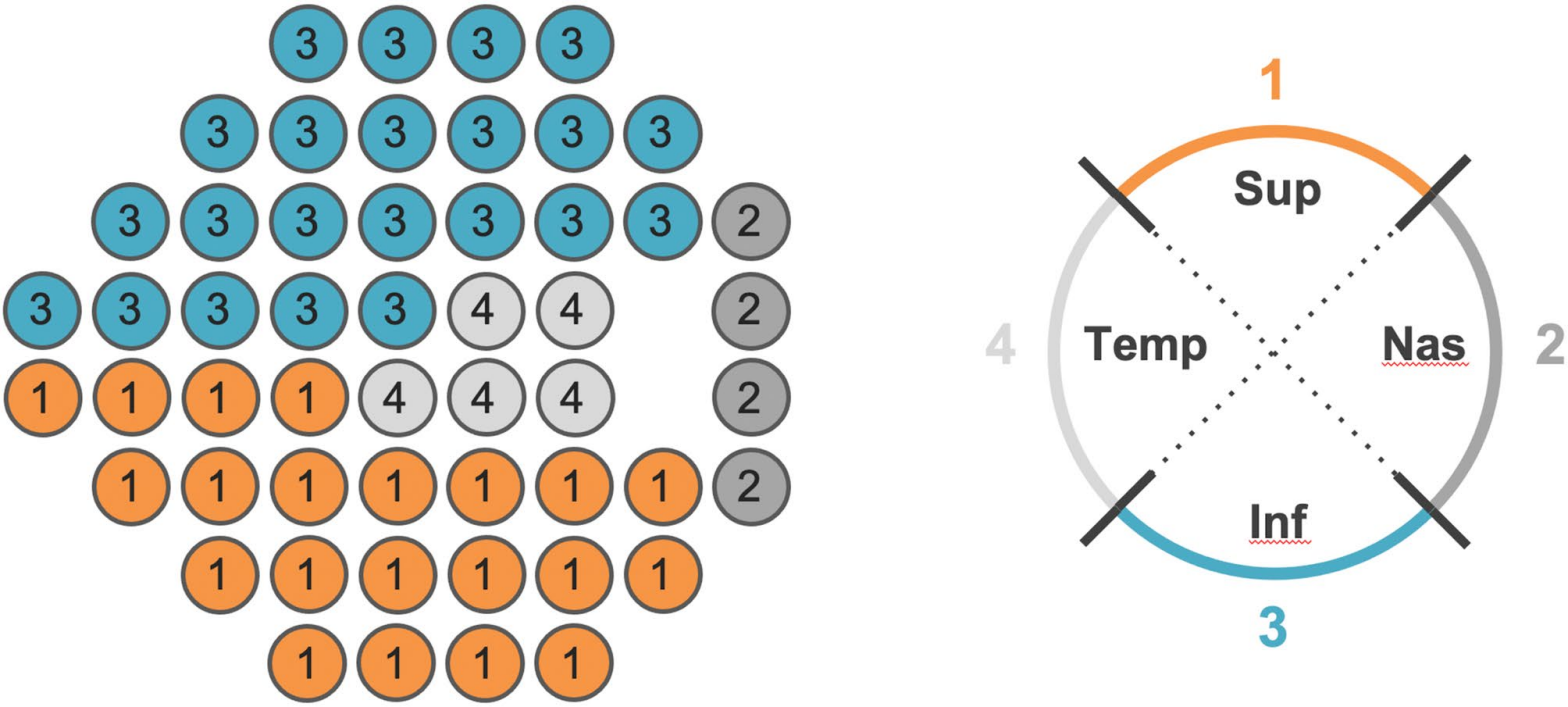
Topographic map relating the stimulus locations on a 24–2 visual field test (left) to regions where the retinal nerve fiber layer thickness was measured on optical coherence tomography (right) is shown for a right eye. *Note*: Sup, Superior; Nas, Nasal; Inf, Inferior; Temp, Temporal.

A simplified combined structure and function index (sCSFI) was developed to combine the structural and functional information from OCT and SAP in order to provide a single estimate of disease severity. First, the topographic relationship between the stimulus locations on the 24–2 visual field test and quadrants of the OCT scan was determined according to a simplification of the map previously proposed by Garway-Heath et al. (Fig. 4)^35^.

The sCSFI combines the structural and functional components through converting both measures into an age-adjusted, linear scale as follows:

$$\text{SAP} = {10}^{[ 0.1 \times \left({\stackrel{-}{ TD}}_{\text{Q}} + 30\right) ]}$$

$$\text{OCT} = {\text{RNFL}}_{{\rm Q}} \, /{\text{ [(b}}_{{\rm Q}}\times \text{ age) + }{\text{c}}_{\text{Q}})] \times{1000}$$

where ${\stackrel{-}{TD}}_{Q}$ represents the average of the total deviation (an age-adjusted parameter) of the points within each quadrant for SAP, and *b*_*Q*_ and *c*_*Q*_ represent the slopes and intercepts of the linear fit between the RNFL thickness and age for the normal eyes at each quadrant (*RNFL*_*Q*_) for OCT using a linear mixed model (see the Statistical Analyses section). In essence, the OCT component consists of the RNFL measurement as fraction of the population-average age-expected value that is multiplied by 1000 (i.e. the value of the SAP component when ${\stackrel{-}{TD}}_{Q}$ = 0 dB). Note that this OCT component should not be interpreted as providing a structural parameter that is equivalent to the SAP component, but merely as a transformed parameter to be used when combining these two parameters in a weighted manner. To combine the SAP and OCT components, a weighting scale similar to the one proposed by Medeiros *et al*^2^ was used in the following manner:

$${\text{sCSFI}}_{{\rm Q}}{ = }(1+{ \stackrel{-}{TD}}_{{\rm Q}}/10) \times \text{OCT} + (-{\stackrel{-}{TD}}_{\text{Q}}/10) \times \text{ SAP}$$

$$s\text{CSFI } = \sum {\text{ sCSFI}}_{{\rm Q}} \times ({\text{ n}}_{{\rm Q}} / {\text{n}}_{{\rm total}} ) / 10$$

$$\text{With }s{\text{CSFI}}_{{\rm Q}} = \text{OCT for } {\stackrel{\mathrm{-}}{\text{TD}}}_{{\rm Q}}\geq{0}$$

$${\text{sCSFI}}_{{\rm Q}} = \text{SAP for } {\stackrel{\mathrm{-}}{\text{TD}}}_{{\rm Q}}\leq-{10}$$

where the sCSFI at each quadrant (CSFI_Q_) consists of the OCT component entirely when ${\stackrel{\mathrm{-}}{\text{TD}}}_{{\rm Q}}\geq{0}$, consists of the SAP component entirely when ${\stackrel{\mathrm{-}}{\text{TD}}}_{{\rm Q}}\leq{ -10}$ and a proportion of each component in between, weighted by ${\stackrel{\mathrm{-}}{\text{TD}}}_{{\rm Q}}$. This weighting scale was chosen to account for the fact that RNFL thickness measurements become asymptotic for visual sensitivities below a total deviation of – 10 dB^5^. The sCSFI is then derived by taking the sum of the sCSFI at each quadrant (sCSFI_Q_) multiplied by the proportion of points sampled in the 24–2 stimulus pattern (*n*_*Q*_) from the total number of stimuli (*n*_*total*_) then divided by 10 to provide a percentage.

### Estimates of retinal ganglion cell counts

The sCSFI was compared to the previous model that required estimating RGC counts. The details of the method for estimating RGC counts have been described previously^2^. The model uses both structural and functional information based on empirical formulas derived by a previous study^4^. The functional component of this model consisted of estimating the number of RGC somas in each retinal area sampled by SAP at eccentricity *e* with a sensitivity *s* in dB is estimated using the following formulas:

$$m=\left[0.054 \times \left(e \times 1.32\right)\right]+0.9$$

$$b=\left[-1.5 \times \left(e \times 1.32\right)\right]-14.8$$

$$gc=\left[\left(s-1\right)-b\right]/ m + 4.7$$

$${SAP}_{rgc}=\sum {10}^{(gc \times 0.1)}$$

The relationship between retinal ganglion cell quantity (*gc*) in decibels and sensitivity (*s*) at each location sampled by SAP is estimated by a linear function, with *m* and *b* representing the slope and intercept respectively. The RGC density at each retinal location sampled by SAP was considered to be uniform over a 6º × 6º area to account for the total number of RGCs in the area. The estimate of RGC count with SAP (*SAP*_*rgc*_) was then obtained by summing all the RGC estimates at all the locations sampled by SAP.

The structural component of this model consisted of estimating the total number of RGC axons present from the average peripapillary RNFL thickness measurements (*RNFL*_avg_) on OCT (*OCT*_*rgc*_). This estimate accounts for the changes in axonal density (*d*, in axons per μm^2^) with age and changes in the composition of the axonal and non-axonal components of the RNFL thickness measurement (using a correction factor *c*) with disease severity (estimated by the visual field mean deviation; MD) with the following formulas:

$$d=\left(-0.007 \times \mathrm{age}\right)+1.4$$

$$c=\left(-0.26 \times \mathrm{MD}\right)+ 0.12$$

$${OCT}_{rgc}={10}^{ [\mathrm{log}\left({RNFL}_{avg} \times 10870 \times d\right) \times (10-c)] \times 0.1}$$

The structural and functional estimates of the RGC number are then combined to provide a single estimate of the RGC count (*eRGC*) using the following weighted scale:

$$eRGC=\left(1+\mathrm{MD}/30\right)\times {OCT}_{rgc}+(-\mathrm{MD}/30) \times {SAP}_{rgc}$$

The rationale for this weighting scale has been described in detail previously^2,3,17^, but in principle is based on the fact that the accuracies of clinical perimetry and imaging tests are inversely related to disease severity.

### Statistical analysis

To convert the OCT imaging derived measurements of RNFL thickness into an age-adjusted component for calculating the sCSFI, linear mixed models (LMMs) were used to determine the relationship between RNFL thickness and age at each quadrant in healthy eyes. LMMs provide a means to account for the correlations present within the hierarchical and repeated-measures nature of the data (since multiple tests from two eyes could be included from each participant). Population-average estimates of the RNFL thickness at a specific age can be determined by using the slopes and intercepts derived from the LMM analyses. To avoid the risk of over-fitting, the parameters of the LMMs were derived using a leave-one-out approach. In essence, the slopes and intercepts of the relationship between RNFL thickness and age at each quadrant were derived for each healthy participant when using all the other healthy participants. In this study, the slopes of the RNFL thickness measurements with age for the superior, temporal, inferior and nasal quadrant were − 0.26, − 0.24, − 0.46 and − 0.13 μm/year respectively and the intercepts were 131, 81, 146 and 73 μm respectively.

The performance of visual field MD, average RNFL thickness, eRGC counts and the sCSFI for discriminating between the glaucoma and normal eyes were evaluated by calculating the AUC, following age-adjustment of the average RNFL thickness and eRGC counts from the values of the healthy participants. The AUCs and standard errors were calculated using a bootstrap resampling procedure (n = 1000 resamples) as previously described, accounting for the correlations within the data structure in this study^36^. The sensitivity for detecting the glaucoma eyes at 95% and 80% specificity were also determined.

LMMs were also used to derive the average and standard deviation (SD) of the normative values for the eRGC count (following age-adjustment) and sCSFI from the healthy eyes, which were then used to convert the eRGC count and sCSFI measurements in the glaucoma eyes into Z-scores (which represent the number of SDs from normal). To calculate the Z-scores, the difference between the eRGC count and sCSFI values from each glaucoma eye and the average normative values were derived, and then divided by the SD of the normative values. The Z-scores of these two parameters were then plotted against each other, and the statistical significance of their difference at 3-dB bins based on visual field MD was determined using LMMs. Comparing the Z-scores provides an opportunity to compare the diagnostic ability of the two parameters quantitatively over the entire spectrum of the disease.

To compare the ability for the average RNFL thickness, eRGC count and sCSFI to stage different levels of glaucomatous visual field damage, AUCs were calculated for the ability of each parameter to discriminate between glaucoma eyes with early (MD > − 6 dB) and moderate (MD − 6 to − 12 dB) visual field loss, and moderate and advanced (MD < − 12 dB) visual field loss. The ability to stage the severity of glaucomatous visual field damage was further evaluated using ordinal logistic regression analyses of each parameter against visual field MD in 3-dB bins. The Nagelkerke pseudo-*R*^2^ was used as the outcome measure of this analysis to provide an estimate of the proportion of explained variance by each parameter, and the pseudo-*R*^2^ and its standard errors were also obtained using a bootstrap resampling procedure (n = 1000 resamples) to account for the correlations within the data structure in this study.
